# Age and gender differences in non-motor symptoms in people with Parkinson’s disease

**DOI:** 10.3389/fneur.2024.1339716

**Published:** 2024-02-01

**Authors:** Bart R. Maas, Irene Göttgens, Hermina P. S. Tijsse Klasen, Willanka M. Kapelle, Danique L. M. Radder, Bastiaan R. Bloem, Bart Post, Nienke M. de Vries, Sirwan K. L. Darweesh

**Affiliations:** ^1^Department of Neurology, Center of Expertise for Parkinson and Movement Disorders, Donders Institute for Brain, Cognition and Behavior, Radboud University Medical Center, Nijmegen, Netherlands; ^2^Department of Primary and Community Care, Radboud Institute for Health Sciences, Radboud University Medical Center, Nijmegen, Netherlands

**Keywords:** Parkinson’s disease, age, gender, non-motor symptoms, quality of life

## Abstract

**Background:**

Non-motor symptoms of Parkinson’s disease (PD) are highly prevalent and heterogenic. Previous studies aimed to gain more insight on this heterogeneity by investigating age and gender differences in non-motor symptom severity, but findings were inconsistent. Furthermore, besides examining the single effects of age and gender, the interaction between them in relation to non-motor functioning has -as far as we know- not been investigated before.

**Objectives:**

To investigate the association of age and gender identity -as well as the interaction between age and gender identity- with non-motor symptoms and their impact on quality of life.

**Methods:**

We combined three large and independent studies. This approach resulted in a total number of unique participants of 1,509. We used linear regression models to assess the association of age and gender identity, and their interaction, with non-motor symptoms and their impact on quality of life.

**Results:**

Older people with PD generally had worse cognitive functioning, worse autonomic functioning and worse quality of life. Women with PD generally experienced more anxiety, worse autonomic functioning and worse quality of life compared to men with PD, whereas men with PD generally had worse cognitive functioning. In interaction analyses by age and gender identity, depressive symptoms and anxiety were disproportionally worse with increasing age in women compared to men.

**Conclusion:**

Our findings indicate that both age and gender -as well as their interaction- are differentially associated with non-motor symptoms of PD. Both research and clinical practice should pay more attention to demographic subgroups differences and possible different treatment approaches with respect to age and gender. We showed how combining datasets is of added value in this kind of analyses and encourage others to use similar approaches.

## 1 Introduction

Parkinson’s disease (PD) is a neurodegenerative disorder characterized by both motor and non-motor symptoms, which affects more than 6 million people worldwide (1). Aging and gender are, together with genetics, environment and immune status, important factors in the development of PD (2) and possibly also in the development of non-motor symptoms. These non-motor symptoms are highly prevalent and often occur years before motor symptoms appear. Non-motor symptoms include anxiety, cognitive dysfunction, depression, autonomic dysfunction and many other symptoms, and they affect quality of life even more than motor symptoms in people with PD (3). Knowledge about non-motor symptoms in PD is very limited compared to motor symptoms (4). Insight into possible subgroup differences could help clinicians to deliver a more person-centred approach (5), informed in part by certain demographic characteristics, such as age and gender.

Previous studies on the associations of age and gender with non-motor symptoms in people with PD did only focus on age and gender as separate factors and not on the interaction between them (6–18). Furthermore, previous findings regarding associations of age and gender with non-motor symptoms were inconsistent. There is evidence that cognitive functioning is worse in men (6, 7), including a large review (18), but some studies found worse cognitive functioning in women with PD (8–10) or no gender differences (11–14). Multiple studies reported a higher burden of depressive symptoms in women with PD (10, 11, 17, 18), but others found no gender differences (12, 16). Higher anxiety levels in women have been reported (14, 17), but others did not find any gender differences (15, 16). There are indications that autonomic dysfunction is more prevalent in women compared to men (8, 13, 14, 17). However, other studies did not find gender differences in autonomic dysfunction (3, 7, 13) or found worse non-motor experiences of daily living in men (19).

Regarding age, previous literature showed conflicting findings with respect to the relationship between age and non-motor symptoms. This is the case for depressive symptoms (20), but also findings on the relationship between age and other non-motor symptoms are inconsistent. One study reported worse cognition, more anxiety and worse autonomic function in older people with PD (6), although two other studies found no relation between age and non-motor symptom severity (3, 8) or less anxiety in older people with PD.

These inconsistent findings might be the result of small sample sizes in some studies, which makes it difficult to draw robust conclusions. Furthermore, differences in selection of covariables, such as anti-depressants, UPDRS and other clinical or demographic variables are also likely to explain discrepant findings. Another potential explanation for inconclusive findings might be that terminology related to sex and gender is often conflated in PD research. Therefore, we used gender identity in this study as a proxy for both potential biological sex-linked differences and sociocultural differences related to gender.

The relationship between non-motor symptoms and quality of life has been investigated, but insight in how this relationship is different between genders or across ages is scarce (12). One could expect considerable differences in personal lives and daily activities of young or middle-aged people with PD, who are often still professionally active or have a (relatively) young family, and older people with PD, for whom the disease introduces different challenges (21). Therefore, non-motor symptoms may impact daily life functioning and thereby quality of life differently for younger people compared to older people with PD.

Taken together, the impact of non-motor symptoms on quality of life may be different across ages and between gender identities. However, this has not been investigated. Therefore, here we investigated the association of the severity of non-motor symptoms with both age and gender identity - as well as with the interaction of age and gender identity - where we hypothesize a greater severity of non-motor symptoms with increasing age. We expect worse cognitive functioning in men, and a higher burden of depressive symptoms, anxiety and worse autonomic function in women. Finally, we investigated whether the association between non-motor symptoms severity and quality of life was different for increasing age and for gender identity. Although we expect that quality of life is better in younger people with PD (22), we hypothesize that non-motor symptoms will have a more negative impact on quality of life for younger people with PD and for women more than for men. These insights will help to further personalize treatment, specifically tailored to gender identity and different age groups.

## 2 Methods

### 2.1 Study design and participants

Baseline data of three large and independent Dutch studies were used, namely the PRIME-NL study (23), Personalized Parkinson Project (PPP) (24) and NICE-PD study (25), all being performed by the Radboudumc Center of Expertise for Parkinson & Movement Disorders based in Nijmegen. PRIME-NL and PPP are observational studies, whereas the NICE-PD is a randomized controlled trial where we only used the baseline data. Inclusion and exclusion criteria for these studies are reported in the study protocols (23–25), but most notably, PPP only included people with PD with a disease duration shorter than 5 years. Participants were recruited in clinics, through ParkinsonNEXT (26) (an online platform to connect people with PD and researchers in the Netherlands), the Dutch Parkinson patient association (27) and Dutch national ParkinsonNet (28) (an existing nationwide clinical infrastructure in the Netherlands with 3,200 specialized PD professionals). In analyses on the combined dataset, overlapping participants between cohorts were removed so that they appeared only once in the entire dataset. These participants were identified based on birthday.

Differences in non-motor symptoms can be the result of biological sex-linked differences, sociocultural differences related to gender or a combination of both. For example, potential differences in cardiovascular dysfunction can to a greater extent be explained by biological sex-linked differences. However, differences in anxiety can be the result of both biological sex-linked differences as well as sociocultural differences related to gender. Therefore, we used gender identity in this study as a proxy for both potential biological sex-linked differences and sociocultural differences related to gender. Gender identity was asked of participants, where “women” and “men” were the only answer options in the PPP and NICE-PD study, while PRIME-NL study also included the answer option “other, namely…”.

The PRIME-NL study has been approved by the Ethical Board of the Radboud University Medical Center. The Personalized Parkinson Project and NICE-PD study have been approved by the local ethics committee (Commissie Mensgebonden Onderzoek Arnhem-Nijmegen). Participants signed a digital or written informed consent before inclusion in the study.

### 2.2 Outcome measures

We included the following non-motor symptoms in our analysis: cognitive functioning, anxiety, depressive symptoms and autonomic dysfunctions. All these measures were assessed in the PRIME-NL study and the PPP. In the NICE-PD study, only autonomic dysfunctions were assessed. Cognitive functioning was assessed using the Telephone Version for the Montreal Cognitive Assessment, with a maximum score of 22 points (T-MoCA). Anxiety was measured with the State–Trait Anxiety Inventory for Adults (STAI). Depressive symptoms were assessed using the Beck Depression Inventory (BDI-II). Autonomic dysfunctions were assessed using the SCales for Outcomes in PArkinson’s Disease – AUTonomic dysfunction (SCOPA-AUT). Total scores on non-motor symptoms questionnaires (STAI, BDI-II and SCOPA-AUT) were the primary endpoints, where domain scores of these questionnaires were secondary endpoints.

### 2.3 Covariates

To correct for potential confounding effects, we corrected for disease duration and study cohort in all analyses. Disease duration was used partly as proxy for motor symptom severity, because data of motor symptoms was not available. Furthermore, we corrected for age and gender identity when it was not the determinant.

### 2.4 Statistical analysis

First, all analyses were done for each study cohort separately, and afterwards for the combined dataset of the three study cohorts.

Primary analysis consisted of multiple linear regression models with both age and gender identity as independent variables and the outcome measure as dependent variable. We determined β-coefficients and *p*-values for each independent variable in each model. We used Bonferroni correction to adjust for multiple-comparison and considered *p* < 0.01 as a significant association with age or significant difference between gender identity.

Secondary analysis consisted of multiple linear regression models with age, gender identity and their interaction [age*gender identity] as independent variables and the outcome measure as dependent variable. We determined β-coefficients and *p*-values for the interaction term and considered *p* < 0.05 as significant evidence for differential association of age on outcome measures for gender identity.

To investigate the impact of non-motor symptoms on quality of life, we computed multiple linear regression models with quality of life as dependent variable, and outcome measures (non-motor symptoms) as independent variables. To investigate whether this impact differed for age and gender identity, we also computed multiple linear regression models with non-motor symptom and age or gender identity and their interaction [non-motor symptom*age] or [non-motor symptom*gender identity] and quality of life as dependent variable.

Statistical analysis was done using R Statistics.

## 3 Results

### 3.1 Socio-demographic characteristics

Table 1 shows the demographic and disease characteristics of the total study population (*n* = 1,509) that consisted of participants in the PRIME-NL study (*n* = 920), PPP (*n* = 519) and NICE-PD study (*n* = 242). In analyses on the combined dataset, 172 participants participated in more than one study and were removed from PRIME-NL (overlap between PRIME-NL and PPP or PRIME-NL and NICE-PD) and/or NICE-PD (overlap between NICE-PD and PPP), so that they appeared only once in the entire dataset. The population consisted of 938 (62%) men and 571 (38%) women with a diagnosis of idiopathic PD. No participants identified as non-binary or gender-divers. The overall mean age of 67.4 years (SD = 9.4) (women 66.6 years; men 67.9 years) and disease duration of 4.6 years (SD = 4.4) (women 4.9 years; men 4.4 years). The youngest participant was 31 years old, and the oldest participant was 93 years old. The mean Hoehn and Yahr stage was 2.2 (SD = 1.0) (women 2.2; men 2.2).

**Table 1:** Characteristics of participants.

	All participants	PRIME-NL study	Personalized Parkinson’s Project	NICE-PD study
	*N = 1,509*	*N = 920*	*N = 519*	*N = 242*
Age (Years)	67.4 (9.4)	69.6 (8.1)	61.7 (9.1)	70.9 (8.1)
Gender (Men)	938 (62.1)	561 (61.0)	306 (59.0)	166 (68.6)
Disease duration (Years since diagnosis)	4.6 (4.4)^a^	5.8 (4.9)^k^	2.7 (1.5)^o^	3.6 (4.0)
Hoehn and Yahr stage	2.2 (1.0)^b^	2.3 (1.2)^l^	1.9 (0.5)^p^	-
Quality of life (PDQ-39, total score)	23.2 (12.7)^c^	25.8 (13.0)	20.5 (11.3)^q^	16.9 (10.3)^t^
Cognitive functioning (T-MoCA)	18.5 (2.8)^d^	18.0 (3.0)^m^	19.5 (2.1)^r^	-
Anxiety (STAI, total score)	75.2 (19.0)^e^	75.6 (18.6)^n^	73.5 (19.3)^s^	-
State-anxiety	37.5 (10.1)^e^	37.6 (10.3)^n^	36.8 (9.6)^s^	-
Trait-anxiety	37.7 (9.7)^e^	38.0 (9.3)^n^	36.6 (10.0)^s^	-
Depressive symptoms (BDI-II)	11.1 (6.8)^e^	11.7 (6.7)^n^	9.8 (6.7)^s^	-
Autonomic dysfunctions (SCOPA-AUT, total score)	14.8 (7.0)^f^	15.8 (7.0)^n^	13.9 (6.7)^s^	12.7 (6.6)^u^
Gastro-intestinal	3.8 (2.7)^g^	4.1 (2.8)^n^	3.4 (2.6)^s^	3.4 (2.5)^v^
Urinary	6.9 (3.6)^h^	7.5 (3.6)^n^	6.6 (3.5)^s^	5.9 (3.5)^w^
Cardiovascular	0.9 (1.2)^i^	1.0 (1.3)^n^	0.8 (1.2)^s^	0.8 (1.2)^x^
Thermoregulatory	2.5 (2.1)^i^	2.6 (2.1)^n^	2.5 (2.1)^s^	2.1 (1.9)^y^
Pupillomotor	0.6 (0.8)^j^	0.7 (0.8)^n^	0.6 (0.8)^s^	0.6 (0.7)^z^

Mean (standard deviation) is presented, except for gender where number of participants (percentage) is presented. Total number of participants (*n* = 1,509) is smaller than sum of number of participants of PRIME-NL study (*n* = 920), Personalized Parkinson’s Project (*n* = 519) and NICE-PD study (242), because based on data of birth there was a possible overlap of 172 participants between studies, which were excluded from the analyses. PDQ-39, Parkinson’s Disease Questionnaire-39; T-MoCA, Telephone Version for the Montreal Cognitive Assessment; STAI; State–Trait Anxiety Inventory for Adults; BDI-II, Beck Depression Inventory; SCOPA-AUT, SCales for Outcomes in PArkinson’s Disease – AUTonomic dysfunction.

^a^*n* = 1,506, ^b^*n* = 1,257, ^c^*n* = 1,442, ^d^*n* = 1,281, ^e^*n* = 1,277, ^f^*n* = 1,443, ^g^*n* = 1,459, ^h^*n* = 1,462, ^i^*n* = 1,461, ^j^*n* = 1,466, ^k^*n* = 919, ^l^*n* = 901, ^m^*n* = 902, *^n^n* = 919, ^o^*n* = 517, ^p^*n* = 492, ^q^*n* = 453, ^r^*n* = 518, ^s^*n* = 497, ^t^*n* = 240, ^u^*n* = 198, ^v^*n* = 215, ^w^*n* = 218, ^x^*n* = 216, ^y^*n* = 217, ^z^*n* = 222.

### 3.2 Associations of age and gender identity with non-motor symptoms

**Figure 1:**
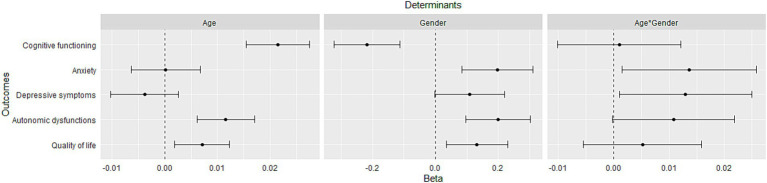
Association between age or gender and non-motor symptoms and quality of life. Points represent the regression coefficients of the linear models and bars the 95% confidence intervals. Outcome measures were standardized in order to make the estimates comparable. Models were adjusted for disease duration and study cohort. Positive β-coefficients for age or gender indicate worse quality of life or worse non-motor functioning for older people or women. Positive β-coefficients for interaction term (age*gender) indicate a worse quality of life or worse non-motor functioning with increasing age for women compared to men, whereas a negative interaction term indicates a worse quality of life or worse non-motor functioning with increasing age for men.

**Figure 2:**
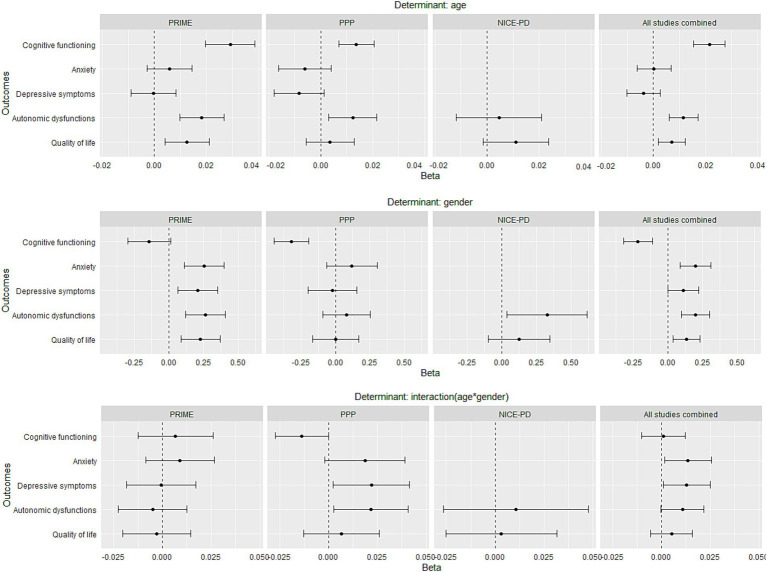
Association between age or gender and non-motor symptoms and quality of life per study cohort (PRIME, PPP, NICE-PD and all studies combined). Points represent the regression coefficients of the linear models and bars the 95% confidence intervals. Outcome measures were standardized in order to make the estimates comparable. Models were adjusted for disease duration and study cohort. Positive β-coefficients for age or gender indicate worse quality of life or worse non-motor functioning for older people or women. Positive β-coefficients for interaction term (age*gender) indicate a worse quality of life or worse non-motor functioning with increasing age for women compared to men, whereas a negative interaction term indicates a worse quality of life or worse non-motor functioning with increasing age for men.

We first ran the primary analysis regarding age for each study cohort separately and then for the combined dataset of the three study cohorts (Figure 1). The single-cohort analysis showed that higher age was associated with worse cognitive functioning in both PRIME and PPP cohorts (Figure 2*, upper graphs*). Anxiety and depressive symptoms were not associated with age in neither the PRIME nor PPP cohorts. Worse autonomic function was associated with higher age in both the PRIME and PPP cohorts, while not in the NICE-PD cohort. After combining the three datasets, higher age was still associated with worse cognitive functioning and with worse overall autonomic functioning (Figure 1*, left graph*), as well as with worse gastro-intestinal-, urinary- and pupillomotor functioning in older people with PD (Supplementary material A*, left graph*). The only exception was thermoregulatory functioning, which was worse in younger people with PD. Cardiovascular dysfunctions were not associated with age.

Regarding gender identity, we first ran the primary analysis for each study cohort separately. This analysis showed that cognitive function was worse in men in the PPP study cohort, while this was not confirmed in the PRIME-NL cohort (β = 0.14, *p* = 0.07) (Figure 2). In the PRIME study, anxiety, depressive symptoms and autonomic functions were all worse in women, while only the latter was found within the NICE-PD cohort and there were no gender differences in these three symptoms in the PPP cohort (Figure 2). After combining datasets, cognitive functioning was worse in men, but women experienced more anxiety and worse overall autonomic functioning (Figure 1*, middle graph*). Within anxiety, both state-and trait-anxiety were more severe in women compared to men (Supplementary material A*, middle graph*). The following autonomic functions were worse in women, compared to men: cardiovascular-, thermoregulatory-and pupillomotor functioning. Gastro-intestinal and urinary dysfunctions did not differ between gender identities.

Secondary analyses were done first for each study cohort separately. Here, most associations of symptom severity with the interaction of age and gender identity pointed (insignificantly) in different directions per study cohort. This was the case for cognitive functioning, depressive symptoms and autonomic dysfunctions (Figure 2). After combining the datasets, this resulted in no significant interaction term, except for depressive symptoms. Anxiety pointed in both PRIME and PPP insignificantly into the same direction, which resulted in a significant interaction term after combining the datasets. The combined analysis thus showed that both anxiety and depressive symptoms are disproportionally more severe with in increasing age for women compared to men (Figure 1*, right graph*), although effect sizes are small, especially for depressive symptoms. In terms of scores on the STAI (anxiety) and BDI-II (depressive symptoms), this means that on average women increase, respectively, with 0.26 points and 0.06 per year of age more compared to men. Within anxiety, only state-anxiety increase was disproportionally more severe with increasing age for women compared to men (Supplementary material A*, right graph*). Within autonomic functions, this was only the case for pupillomotor dysfunctions, and other sub scores were not significantly associated with the interaction between age and gender identity.

### 3.3 Associations of age and gender identity with quality of life

Higher age and being a woman were both associated with a lower quality of life in only the PRIME cohort, while this association was not significant in the PPP and NICE-PD cohort (Figure 2). After combining the three datasets, these associations were significant (Figure 1). The decrease of quality of life with increased age was not different for men or women with PD (Figure 1).

### 3.4 Associations of non-motor symptoms with quality of life

Higher severity of all non-motor symptoms was associated with lower quality of life (Supplementary material B*, left graph*). Age or gender identity did not have an impact on this association between non-motor symptoms and quality of life (Supplementary material B*, middle and right graph*).

Unstandardized differences between gender identity or age on all outcome measures are shown in Supplementary material C.

## 4 Discussion

This is -as far as we know- the first study that investigated the association of the interaction between age and gender identity with non-motor symptoms, which informs us whether non-motor symptoms are disproportionally worse with increase of age for women or men with PD. We found that both anxiety and depressive symptoms, but not autonomic symptoms, are disproportionally worse with increasing age for women compared to men with PD. These findings on group level are clinically relevant to facilitate a more individually tailored treatment of people with PD by raising more awareness in clinicians.

Regarding the associations between gender and non-motor symptoms, most of our findings were in line with our hypotheses and previous evidence, including worse cognitive functions in men (6, 7, 18) and more severe anxiety (14, 17), autonomic dysfunctions (8, 13, 14, 17) and worse quality of life in women with PD. The only exception was that we did not find a higher burden of depressive symptoms in women (β = 0.11, *p* = 0.056).

Also, most of our findings regarding associations of age with non-motor symptoms were in line with our hypotheses, including worse cognitive functions, autonomic functions and quality of life with increasing age. The associations of higher age with worse cognitive functioning and worse autonomic functions were to be expected and in line with previous studies (6). Our hypothesis was not confirmed for anxiety and depression were we did not find an effect of age. The association of age with anxiety and depressive symptoms is less clear (20), which may reflect differences between studies in the age span of participants, as these symptoms are sometimes worse in people with young-onset PD.

Our insights will help to further personalize treatment specifically for men and women and different age groups, because it guides clinicians on which symptoms to pay particular attention to. Inconsistent findings of previous studies regarding both age and gender identity can be explained by the smaller sample size (3, 12) and the younger age of participants (15, 29), whereas our study participants are (with a mean age of 67 years and 38% women) are more representative of the full PD population (with a mean age of 72 years and 42% women) (30).

The relationship between non-motor symptoms and quality of life has been investigated, but insight in how this relationship is different between genders is scarce (12) and therefore our analyses were rather exploratory. One small previous study (n = 89) found that higher levels of depressive symptoms were associated with lower quality of life in women, but not in men with PD (12). However, this previous finding might be explained by the low sample size of men (n = 47) (12). In the present study, we did not find a differential impact of non-motor symptoms on quality of life across ages and between genders. There might be several explanations for not finding this effect. First, we might lack statistical power to detect the non-linear effects of age. Although we hypothesized that the quality of life of younger people with PD is better compared to older people with PD, in line with findings in people with young-onset PD (22), we expected the impact of non-motor symptoms on quality of life to be larger for younger people with PD. To illustrate this possible effect, the impact of being incontinent may probably have a larger impact on the quality of life of a younger person, because of a professionally active or has a young family, compared to someone who is retired and at home most of the day. However, people with young-onset PD (diagnosis of PD < 40 years), in whom the impact of (non-motor) symptoms on quality of life is probably the largest, comprised only 1.7% of the participants (n = 26). Another possible explanation for not finding a differential impact of non-motor symptoms on quality of life across ages and between genders, may be the wide range of topics that the PDQ-39 covers and therefore the instrument may not be able to detect specific interactions. Although we did not find a differential impact of non-motor symptoms on quality of life across ages and between genders, all the non-motor symptoms that were studied negatively impacted quality of life. This finding highlights the burden of those non-motor symptoms and the importance of identification and treatment of the symptoms.

A unique aspect of the current study is that we were able to combine datasets of multiple studies using similar methodology, which increased our sample size. At our research centre (Radboudumc Center of Expertise for Parkinson & Movement Disorders in Nijmegen, the Netherlands), more than 30 datasets of unique study cohorts are collected or still being collected (30). Combining multiple datasets enabled us to perform regression analyses including interaction terms, for which more statistical power is needed than for investigating a single association. This is highlighted in Figure 1, where we show that determinants in single-study analyses insignificantly point in the same direction in all studies but become significant determinants after combining datasets (for example on anxiety). This example shows the added value of combining datasets of multiple studies, which may be useful for other research groups that aim to investigate associations of clinical measures and especially the interaction between them. These interaction analyses would not be possible by meta-analysing summary statistics of previous studies. Another benefit of combining datasets might be the investigation of associations between a variety of measures that are often not all assessed in a single study, including genetics, imaging, clinical-or questionnaire data. In addition, reduction of costs may be another benefit, by not having to repeat an expensive measure like Magnetic Resonance Imaging for participants that recently had such measure in another study. However, there are also factors that researchers should consider when combining datasets. First, the statistical power increases by increasing the number of participants and this may result in statistically significant findings that are not clinically relevant. Researchers should thus pay attention to the effect sizes. Second, the privacy of study participants should be the number one priority, and combining datasets potentially increases the risk of de-identification. Therefore, researchers must check whether they are allowed to combine datasets of participants that participate in multiple studies and take appropriate protective measures accordingly.

Decline of non-motor functioning, related to aging also occurs in the healthy population and also here differences between women en men are found (8). These differences can be explained by differences in social gender dimensions, but also by biological sex-linked differences. Regarding sex differences, the protective role of oestrogens in women (2) and the decrease in oestrogen levels in women during and after the menopause may play a role in autonomic functions (31). The decrease of oestrogen levels are associated with a peak of autonomic dysfunction during the menopause in healthy women (32). These autonomic dysfunctions include disorders of thermoregulation (33), cardiovascular disease (31), sexual dysfunction (31) and urinary incontinence (32). On average, the menopause presents on the age of 51 until 58 years (31). These autonomic dysfunctions then steadily increase after the menopause in healthy women (32). In our study, where we assume that most participants of our sample are post-menopause, we found similar results. Besides these biological sex differences, differences in non-motor symptoms may also be explained by gender dimensions (34). An example of this is that regardless of gender identity, higher traditional masculine gender roles predict lower anxiety and depressive symptoms (35). It seems that both women and elderly are more at-risk for developing non-motor symptoms, regardless of having PD or not (5). The finding of worse cognitive functioning in men with PD seems to be specific to PD, although no explanation for this association exists (5). Furthermore, in people with PD, both the incidence and severity of non-motor symptoms are higher compared to age-matched healthy controls (8, 11, 15, 17). It is still unknown whether there is an interplay between degeneration of the dopaminergic system, ‘normal’ influence of aging and oestrogen levels. Further research should focus on the underlying social and biological mechanisms to better understand the, possibly differential, pathophysiology of non-motor symptoms in women with PD.

The current study also has some limitations. Specifically, measures of non-motor symptoms were self-reported, which means that interindividual differences in the accuracy and ability to cope with non-motor symptoms in their daily life may have influenced the results. Future research should therefore also include measures to objectively evaluate the severity of non-motor symptoms, in particular those related to autonomic symptoms. Another limitation of our study is that we were not able to correct for the severity of motor symptoms across all cohorts, because non-motor symptom severity was not assessed in all studies. Although we are aware that disease duration does not perfectly correlate with motor symptom severity, we used disease duration as a proxy for motor symptoms to correct for this in the pooled analysis across all cohorts. Furthermore, we performed a sensitivity analysis in single-cohort datasets where we corrected for motor symptom severity (UPDRS-III score), when available (PPP and NICE-PD cohorts). Adding the motor symptom severity in these single study sensitivity analyses did not substantially change the results of the main analyses without this variable. Results of this sensitivity analysis are shown in Supplementary material D. Other potential cofounding factors, such as cognition, were not assessed in all three cohorts or were assessed in a different way across cohorts. To exclude the potential confounding effect of cognition on depressive symptoms and vice versa, we performed a sensitivity analysis in which we corrected for depressive symptoms when cognition was the outcome measure and vice versa. We did this in single-cohort datasets where MoCA was available (PRIME-NL and PPP). Adding the cognitive function or depressive symptoms in single study sensitivity analyses did not substantially change the results of the main analyses without these covariates. Results of this sensitivity analysis are shown in Supplementary material E. Future research could also investigate age and gender identity differences in other non-motor symptoms, including sleeping problems, hallucinations and apathy.

Nowadays, care for people with PD is moving from a one-size-fits-all approach towards a more personalized approach (36). Although our group-level findings do not directly inform the care of one person with PD, identifying social and biological characteristics of people that have a higher propensity to develop certain symptoms may contribute to personalized care. Raising awareness in clinicians on these group-level differences may lead to earlier identification and thereby earlier and more personalized treatment. We showed how combining datasets is of added value in this kind of analyses and encourage others to use similar approaches.

## Data availability statement

The raw data supporting the conclusions of this article will be made available from the corresponding author on reasonable request. Requests to access these datasets should be directed to Sirwan.darweesh@radboudumc.nl.

## Ethics statement

The PRIME-NL study has been approved by the Ethical Board of the Radboud University Medical Center. The Personalized Parkinson Project and NICE-PD study have been approved by the local ethics committee (Commissie Mensgebonden Onderzoek Arnhem-Nijmegen). The studies were conducted in accordance with the local legislation and institutional requirements. The participants provided their written informed consent to participate in this study.

## Author contributions

BM: Conceptualization, Data curation, Formal analysis, Visualization, Writing – original draft. IG: Conceptualization, Supervision, Writing – review & editing. HK: Formal Analysis, Writing – original draft. WK: Writing – review & editing. DR: Data curation, Writing – review & editing. BB: Funding acquisition, Writing – review & editing. BP: Writing – review & editing. NV: Supervision, Writing – review & editing. SD: Conceptualization, Supervision, Writing – review & editing.
