# Supplementary material for: Age and gender differences in non-motor symptoms in people with Parkinson’s disease
Source: Front Neurol. 2024 Feb 1;15:1339716. doi: 10.3389/fneur.2024.1339716 (PMC10867965; doi:10.3389/fneur.2024.1339716)
Supplement: Supplementary file 1 [file Data_Sheet_1.pdf]

Supplementary Material

Age and gender differences in non-motor symptoms in people with Parkinson's disease

Bart R. Maas[1], MSc, Irene Göttgens[2], MSc, Hermina P.S. Tijsse Klasen[1], BSc, Willanka M. Kapelle[1], MD, Danique L.M. Radder[1], MD, Bastiaan R. Bloem[1], MD, PhD, FRCPE, Bart Post[1], MD, PhD, Nienke M. de Vries[1], PhD, Sirwan K.L. Darweesh[1*], MD, PhD

*** Correspondence:** Sirwan K.L. Darweesh: Sirwan.Darweesh@radboudumc.nl

1 Supplementary Figures and Tables

Supplementary material

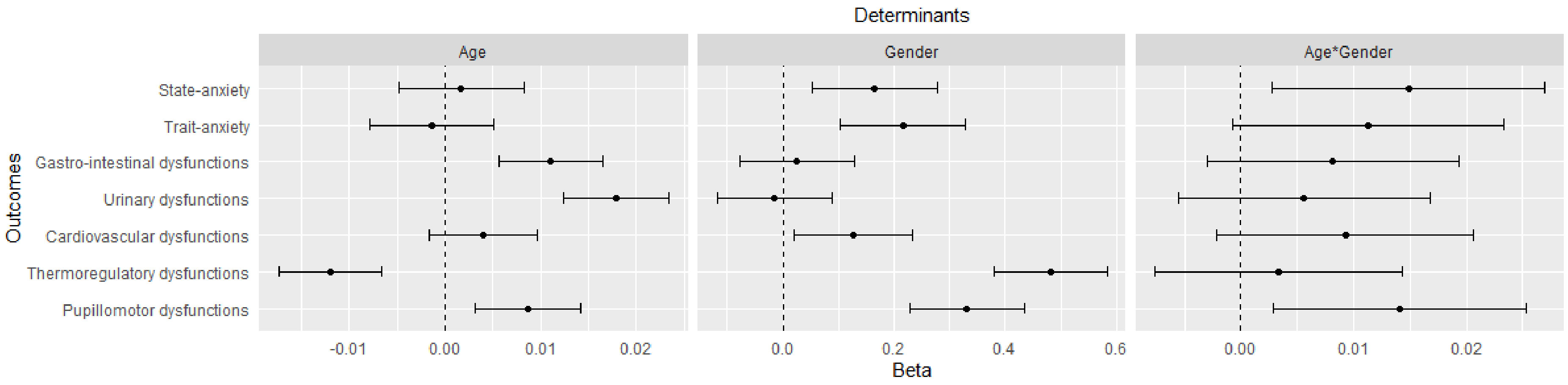

Supplementary material A. Association between age or gender and domain scores of non-motor symptoms. Points represent the regression coefficients of the linear models and bars the 95% confidence intervals. Non-motor outcomes were standardized in order to make the estimates comparable. Models were adjusted for disease duration and study cohort. Positive β-coefficients for age or gender indicate worse quality of life or worse non-motor functioning for older people or women. Positive β-coefficients for interaction term (age*gender) indicate a worse quality of life or worse non-motor functioning with increasing age for women compared to men, whereas a negative interaction term indicates a worse quality of life or worse non-motor functioning with increasing age for men.

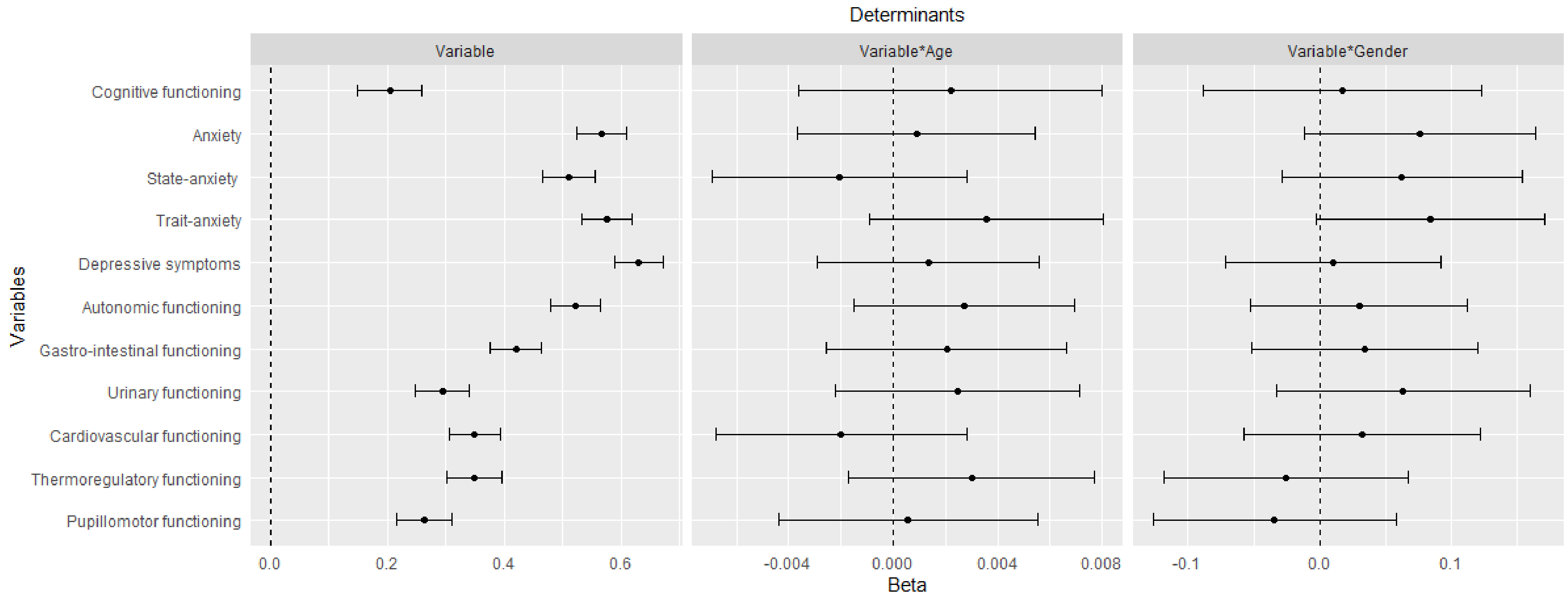

Supplementary material B. Impact of non-motor symptoms on quality of life. Points represent the regression coefficients of the linear models and bars the 95% confidence intervals. Non-motor outcomes and quality of life were standardized in order to make the estimates comparable. Models were adjusted for age, gender, disease duration and study cohort. Positive β-coefficients for variables indicate that worse non-motor functioning was associated with worse quality of life. Positive β-coefficients for interaction term (Variable*Age or Variable*Gender) indicate that non-motor symptoms have a larger impact on quality of life with increased age or for women.

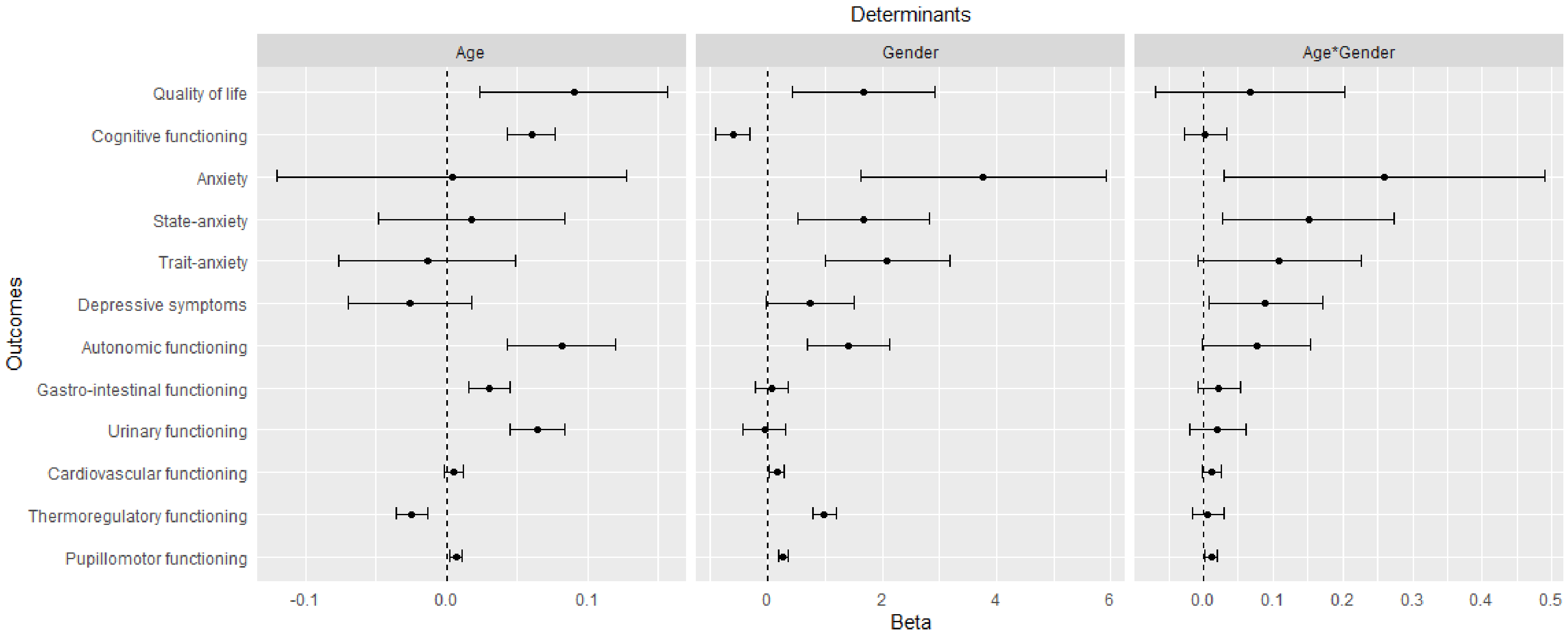

Supplementary material C. Association between age or gender and quality of life and non-motor symptoms. Points represent the regression coefficients of the linear models and bars the 95% confidence intervals. β-coefficients indicate de increase/decrease on outcome measures per increased year of age or difference between genders. Models were adjusted for disease duration and study cohort. Positive β-coefficients for age or gender indicate worse quality of life or worse non-motor functioning for older people or women. Positive β-coefficients for interaction term (age*gender) indicate a worse quality of life or worse non-motor functioning with increasing age for women compared to men, whereas a negative interaction term indicates a worse quality of life or worse non-motor functioning with increasing age for men.

Supplementary material D. Sensitivity analysis with correction for motor symptom severity					
		**Cohort**			
		**PPP**		**NICE-PD**	
**Determinant**	**Dependent variable**	**Without correction**	**With correction**	**Without correction**	**With correction**
Age	Cognitive functioning	0.014 [0.007; 0.020]***	0.016 [0.006; 0.026]**		
	Anxiety	-0.006 [-0.016; 0.004]	-0.010 [-0.021; 0.001]		
	Depressive symptoms	-0.008 [-0.018; 0.001]	-0.013 [-0.024; -0.001]*		
	Autonomic dysfunctions	0.012 [0.003; 0.022]**	0.006 [-0.005; 0.017]	0.005 [-0.012; 0.021]	0.000 [-0.018; 0.018]
	Quality of life	0.004 [-0.006; 0.013]	-0.001 [-0.012; 0.010]	0.011 [-0.001; 0.024]	0.000 [-0.014; 0.014]
Gender	Cognitive functioning	-0.319 [-0.444; -0.193]***	-0.420 [-0.610; -0.230]***		
	Anxiety	0.120 [-0.063; 0.304]	0.160 [-0.042; 0.362]		
	Depressive symptoms	-0.022 [0.198; -0.155]	0.093 [-0.108; 0.294]		
	Autonomic dysfunctions	0.081 [-0.089; 0.251]	0.126 [-0.067; 0.319]	0.330 [0.040; 0.620]*	0.349 [0.029; 0.668]*
	Quality of life	0.002 [-0.165; 0.168]	0.081 [-0.118; 0.280]	0.126 [-0.095; 0.348]	0.125 [-0.123; 0.373]
Age*Gender	Cognitive functioning	-0.014 [-0.027; -0.000]*	-0.024 [-0.045; -0.003]*		
	Anxiety	0.019 [-0.002; 0.039]	0.011 [-0.013; 0.034]		
	Depressive symptoms	0.022 [0.002; 0.042]*	0.014 [-0.009; 0.037]		
	Autonomic dysfunctions	0.022 [0.003; 0.041]*	0.019 [-0.004; 0.041]	0.011 [-0.027; 0.048]	0.003 [-0.036; 0.042]
	Quality of life	0.007 [-0.013; 0.026]	0.006 [-0.019; 0.030]	0.001 [-0.025; 0.032]	-0.009 [-0.040; 0.021]

*p<0.05, **p<0.01, ***p<0.001

Beta-coefficients [95% confidence interval] are presented. All variables are corrected for disease duration. We additionally corrected for motor symptom severity (With correction) or not (Without correction).

**Supplementary material E. Sensitivity analyses with correction for cognitive functioning or depressive symptoms.**					
		**Cohort**			
		**PRIME**		**PPP**	
**Determinant**	**Dependent variable**	**Without correction**	**With correction**	**Without correction**	**With correction**
Age	Cognitive functioning	0.029 [0.020; 0.039]***	0.029 [0.020; 0.039]***	0.014 [0.007; 0.020]***	0.015 [0.008; 0.022]***
	Depressive symptoms	-0.0003 [-0.009; 0.008]	-0.006 [-0.015; 0.003]	-0.008 [-0.018; 0.001]	-0.009 [-0.019; 0.000]
Gender	Cognitive functioning	-0.143 [-0.299; 0.013]	-0.179 [-0.334; -0.025]*	-0.319 [-0.444; -0.193]***	-0.334 [-0.462; -0.205]***
	Depressive symptoms	0.210 [0.065; 0.354]**	0.221 [0.077; 0.364]**	-0.022 [0.198; -0.155]	0.005 [-0.176; 0.186]
Age*Gender	Cognitive functioning	0.007 [-0.012; 0.026]	-0.013 [-0.028; 0.001]	-0.014 [-0.027; -0.000]*	-0.013 [-0.028; 0.001]
	Depressive symptoms	-0.001 [-0.018; 0.017]	0.000 [-0.011; 0.025]	0.022 [0.002; 0.042]*	0.021 [0.002; 0.040]*

*p<0.05, **p<0.01, ***p<0.001

Beta-coefficients [95% confidence interval] are presented. All variables are corrected for disease duration. When cognitive functioning was the dependent variable, we additionally corrected for depressive symptoms (With correction) or not (Without correction). When depressive symptoms was the dependent variable, we additionally corrected for cognitive functioning (With correction) or not (Without correction).
